# Quality and utility of [^123^I]I-metaiodobenzylguanidine cardiac SPECT imaging in nondiabetic postinfarction heart failure patients qualified for implantable cardioverter defibrillator

**DOI:** 10.1007/s12149-021-01628-1

**Published:** 2021-05-23

**Authors:** Anna Teresińska, Olgierd Woźniak, Aleksander Maciąg, Jacek Wnuk, Jarosław Jezierski, Aneta Fronczak, Elżbieta Katarzyna Biernacka

**Affiliations:** National Institute of Cardiology, Alpejska 42, 04-628 Warsaw, Poland

**Keywords:** MIBG cardiac imaging, Single-photon emission computed tomography, SPECT, Heart failure

## Abstract

**Objective:**

Impaired cardiac adrenergic activity has been demonstrated in heart failure (HF) and in diabetes mellitus (DM). [^123^I]I-metaiodobenzylguanidine (MIBG) enables assessment of the cardiac adrenergic nervous system. Tomographic imaging of the heart is expected to be superior to planar imaging. This study aimed to determine the quality and utility of MIBG SPECT in the assessment of cardiac innervation in postinfarction HF patients without DM, qualified for implantable cardioverter defibrillator (ICD) in primary prevention of sudden cardiac death.

**Methods:**

Consecutive patients receiving an ICD on the basis of contemporary guidelines were prospectively included. Planar MIBG studies were followed by SPECT. The essential analysis was based on visual assessment of the quality of SPECT images (“high”, “low” or “unacceptable”). The variables used in the further analysis were late summed defect score for SPECT images and heart-to-mediastinum rate for planar images. MIBG images were assessed independently by two experienced readers.

**Results:**

Fifty postinfarction nondiabetic HF subjects were enrolled. In 13 patients (26%), the assessment of SPECT studies was impossible. In addition, in 13 of 37 patients who underwent semiquantitative SPECT evaluation, the assessment was equivocal. Altogether, in 26/50 patients (52%, 95% confidence interval 38–65%), the quality of SPECT images was unacceptable or low and was limited by low MIBG cardiac uptake and by comparatively high, interfering MIBG uptake in the neighboring structures (primarily, in the lungs).

**Conclusions:**

The utility of MIBG SPECT imaging, at least with conventional imaging protocols, in the qualification of postinfarction HF patients for ICD, is limited. In approximately half of the postinfarction HF patients, SPECT assessment of cardiac innervation can be impossible or equivocal, even without additional damage from diabetic cardiac neuropathy. The criteria predisposing the patient to good-quality MIBG SPECT are: high values of LVEF from the range characterizing the patients qualified to ICD (i.e., close to 35%) and left lung uptake intensity in planar images comparable to or lower than heart uptake.

## Introduction

Metaiodobenzylguanidine is a chemical noradrenaline analogue, which, after labeling with a ^123^I radioisotope, enables scintigraphy assessment of neuronal integrity and function in the cardiac adrenergic nervous system (CANS) [1]. Heart failure (HF) is closely related to the dysfunction of CANS [2]. Reports have described the prognostic relevance of evaluating the planar images of [^123^I]I-metaiodobenzylguanidine (MIBG) heart uptake in HF patients, and it is expected that a three-dimensional technique may improve prognostic strength [3–6]. MIBG imaging with single-photon emission computed tomography (SPECT) is expected to predict the inducibility of ventricular tachycardia [7] and adequate therapy of an implantable cardioverter defibrillator (ICD) [8, 9]. Denervation hypersensitivity, which results in ventricular tachycardia [10], may be caused by regional disorders in the myocardial innervation system that are induced by infarction, and the SPECT technique has a potential to identify denervated myocardial regions [11, 12].

Beyond HF, impaired cardiac MIBG uptake is demonstrated in diabetes mellitus (DM) patients [13–20]. The prevalence of diabetic cardiac neuropathy in patients with type 2 DM is estimated to be approximately 20–36% [18, 21, 22].

Therefore, in ischemic HF patients with DM, the disorders caused by diabetic neuropathy can confound the effect of ischemia on cardiac MIBG uptake. The results of studies performed without the exclusion of patients with DM are dependent on the proportion of DM patients and do not address, in an isolated manner, the state of the CANS that develops after infarction. According to our knowledge, none of the published literature has analyzed MIBG SPECT quality in a homogenous group of patients that: (1) have postinfarction HF and (2) do not have DM.

This work aimed to determine the quality and utility of MIBG SPECT imaging to assess the cardiac innervation in HF patients, postinfarction, without DM, who were qualified for an ICD for the primary prevention of sudden cardiac death (SCD).

## Materials and methods

### Patient population

This work was the first stage of a single-center, prospective project. The project is composed of consecutive patients with postinfarction HF that qualified for implantation of an ICD in primary prevention of SCD, complemented with cardiac resynchronization therapy (CRT-D) if indicated, on the basis of the guidelines for management of HF [23, 24]. Qualified patients are symptomatic (NYHA Class II–III), with left ventricular ejection fraction (LVEF) ≤ 35%, > 40 days after myocardial infarction, ≥ 3 months on optimal medical therapy, expected to survive substantially longer with good functional status. Exclusion criterion for the project is age < 50 years which was entered to omit the potential problem of pregnancy in female patients, and which was equally applied to men to limit inhomogeneity in the studied population. For this paper, the exclusion criterion was DM. MIBG scintigraphy had been performed within 3 months before or after an ICD or CRT-D implantation (and without defibrillation between the time of device implantation and the MIBG study). The project was approved by the Regional Bioethics Committee (Permission Number 1277). Informed signed consent was obtained from all the participants prior to the MIBG procedure.

### Imaging protocol

None of the patients was taking medications that would affect organ uptake of MIBG. At least 24 h before MIBG injection, patients were instructed to stop eating food and compounds that could influence MIBG distribution [25].

MIBG was obtained from a commercial source (National Centre for Nuclear Research—Radioisotope Centre POLATOM, Poland). MIBG activity of 3.8–10.4 mCi (141–384 MBq) was intravenously administered over 2 min [25]. 10 min long planar images of the heart were acquired 3.5 h after MIBG injection [25] and were followed by a 30 min long session of SPECT imaging. A dual-head gamma camera (AXIS, Picker/Philips) equipped with low-energy high-resolution (LEHR) collimators was utilized, with a 20% energy window centered on the 159 keV I-123 photopeak. Planar images were obtained from an anterior thoracic view in a 128 × 128 matrix. In the SPECT studies, 68 projections in a 64 × 64 matrix were acquired from the right anterior oblique to left posterior oblique view (180º) [25]. The SPECT data were reconstructed through filtered back projection (FBP) after applying a two-dimensional Butterworth filter (cutoff frequency of 0.4 cycles/cm and the order of 7). Standard short-axis left ventricular (LV) images, horizontal and vertical long-axis images, as well as a polar map of radiopharmaceutical distribution in the LV, were obtained through Emory Cardiac Toolbox software.

As the uptake and washout of MIBG from liver and lungs are specific in HF, early-phase imaging might provide better visual evaluation SPECT images graded as ‘unacceptable’ and/or ‘low’ quality in late phase imaging. To discuss that aspect of MIBG SPECT, the early-phase images, available in some but not in all the 50 patients, acquired and reconstructed according to the same protocol as in late phase, were used.

### Image analysis

Image processing and analysis was performed on an ODYSSEY workstation (Picker/Philips) by two observers. Observer 1 was a nuclear medicine specialist with 30 years of experience, including 15 years in nuclear cardiology. Observer 2 was a medical physics specialist, with 30 years of experience in nuclear cardiology image acquisition, processing and methods of assessment.

SPECT image processing was completed by Observer 2 and analysis of reconstructed data was performed by both observers during independent sessions. Each reconstructed SPECT image was assessed visually. A scale of zero to two was utilized to evaluate the quality of the images. Two, or “high”, was assigned when good-quality images enabled a reliable assessment of the study. One, or “low”, was assigned when high extracardiac uptake interfered with the uptake of MIBG in the LV wall(s), leading to an equivocal assessment of the study. Zero, or “unacceptable”, was assigned when very high extracardiac uptake that was contiguous with the LV wall(s) precluded assessment of the study. If the grade was 0 from at least one observer, the study was excluded from semiquantitative evaluation (the group SPECTnonquant). In the remaining studies, semiquantitative assessment of MIBG uptake was performed (the group SPECTquant) with a standardized 17-segment model of the LV [25]. A score was assigned to the tracer uptake in each segment by a 5-point scale (0 = normal, 1 = mildly reduced, 2 = moderately reduced, 3 = severely reduced, 4 = absent). A total score was calculated by adding the scores from each segment. The mean total score that was achieved by the observers was the summed defect score (SDS) for the patient. The available early SPECT images were reviewed by single observer (Observer 2).

From planar images, global MIBG cardiac uptake was evaluated through the heart-to-mediastinum ratio (HMR) [25]: the counts per pixel in the myocardium (H) were calculated from the region of interest (ROI) circumscribing the heart. The counts per pixel in the mediastinum (M) were calculated from 7 × 7 pixels in the ROI in the upper mediastinum, avoiding the thyroid gland. Two observers conducted planar image processing and analysis independently. The H/R value was calculated by each observer. The mean of the H/R values calculated by the two observers was the HMR value for the patient.

Additional consensus qualitative assessment of planar MIBG images, performed by both observers on separate occasion after finishing all the individual assessments, was conducted. The relative uptake intensity among heart, lung and liver was qualified as “higher than”, “comparable to” or “lower than” and connected with groups of SPECT results (SPECTnonquant, SPECTquant, SPECTlow, and SPECThigh).

### Statistical analysis

Continuous variables were expressed as means ± standard deviation and ranges, and categorical variables were expressed as percentages. A 95% confidence interval (CI_0.95_ < from–to >) for the frequency of events was calculated for a binominal distribution. As a test of normality, the Kolmogorov–Smirnov test was utilized. The relationship between the HMR values from planar studies and the SDS values from the SPECT studies was assessed through linear regression analysis and calculation of the Pearson correlation coefficient (*R*). The coefficient of variation of repeated measurements (CV%) was used to evaluate the intra-observer and the inter-observer reproducibility [26]. The significance level for statistical tests was set at *p* < 0.05. Statistical analyses were performed using SAS v.9.4 for Windows 7 Professional × 64 (SAS Institute Inc., Cary, NC, USA).

## Results

Fifty nondiabetic postinfarction HF patients, who qualified for an ICD for the primary prevention of SCD, were enrolled. The characteristics of the patients are summarized in Table 1 and the parameters for MIBG imaging are presented in Table 2.

**Table 1:** Characteristics of the included patients (*n* = 50)

Characteristics	Value
Male gender	43 (86)
Age (years)	67 ± 14 (50–84)
Weight (kg)	80 ± 14 (52–120)
Height (cm)	171 ± 7 (158–186)
NYHA functional class	
II	37 (74)
II/III	4 (8)
III	9 (18)
Number of myocardial infarctions per patient	1.5 ± 0.7 (1–3)
Time of MIBG imaging in relation to last myocardial infarction (months)	94 ± 82 (3–336)
Patients with history of percutaneous coronary interventions PCI	33 (66)
Patients with history of coronary artery bypass grafting CABG	13^a^ (26)
Patients with ICD implanted ≤ 3 months before MIBG imaging	22 (44)
Patients with CRT-D implanted ≤ 3 months before MIBG imaging	6 (12)
Patients with ICD implanted ≤ 3 months after MIBG imaging	17 (34)
Patients with CRT-D implanted ≤ 3 months after MIBG imaging	5 (10)
LVEF (%)	27 ± 5 (13–35)
Hypertension	23 (46)
Dyslipidaemia	25 (50)
Medications	
Beta blocker	50 (100)
Angiotensin-converting enzyme inhibitor	40 (80)
Angiotensin receptor blockers	7 (14)
Loop diuretics	41 (82)
Aldosterone antagonist	18 (36)

Values are means ± SD (range) or number (%)

*CABG* coronary artery bypass grafting, *CRT-D* cardiac resynchronization therapy, *ICD* implantable cardioverter defibrillator, *LVEF* left ventricular ejection fraction, *MIBG* [^123^I]I-metaiodobenzylguanidine, *NYHA* New York Heart Association, *PCI* percutaneous coronary intervention

^a^The group of 13 patients with history of CABG included 5 patients with history of PCI

**Table 2:** Characteristics of MIBG imaging in 50 included patients

Characteristics	Value
Administered activity per kg of body weight (MBq/kg)	4.3 ± 0.7 (2.1–5.3)
Total administered activity (MBq)	338 ± 43 (141–384)
Planar imaging after MIBG injection (h)	3.62 ± 0.22 (3.37–4.25)
SPECT imaging after MIBG injection (h)	4.00 ± 0.58 (3.67–6.58)
HMR in the whole group of 50 patients	1.58 ± 0.23 (1.00–2.15)
HMR in 37 patients submitted to semiquantitative SPECT assessment	1.62 ± 0.20 (1.00–1.97)
SDS in 37 patients submitted to semiquantitative SPECT assessment	34 ± 11 (6–68)

Values are means ± SD (range)

*HMR* heart-to-mediastinum ratio, *MIBG* [^123^I]I-metaiodobenzylguanidine, *SDS* summed defect score, *SPECT* single-photon emission computed tomography

**Fig. 1:**
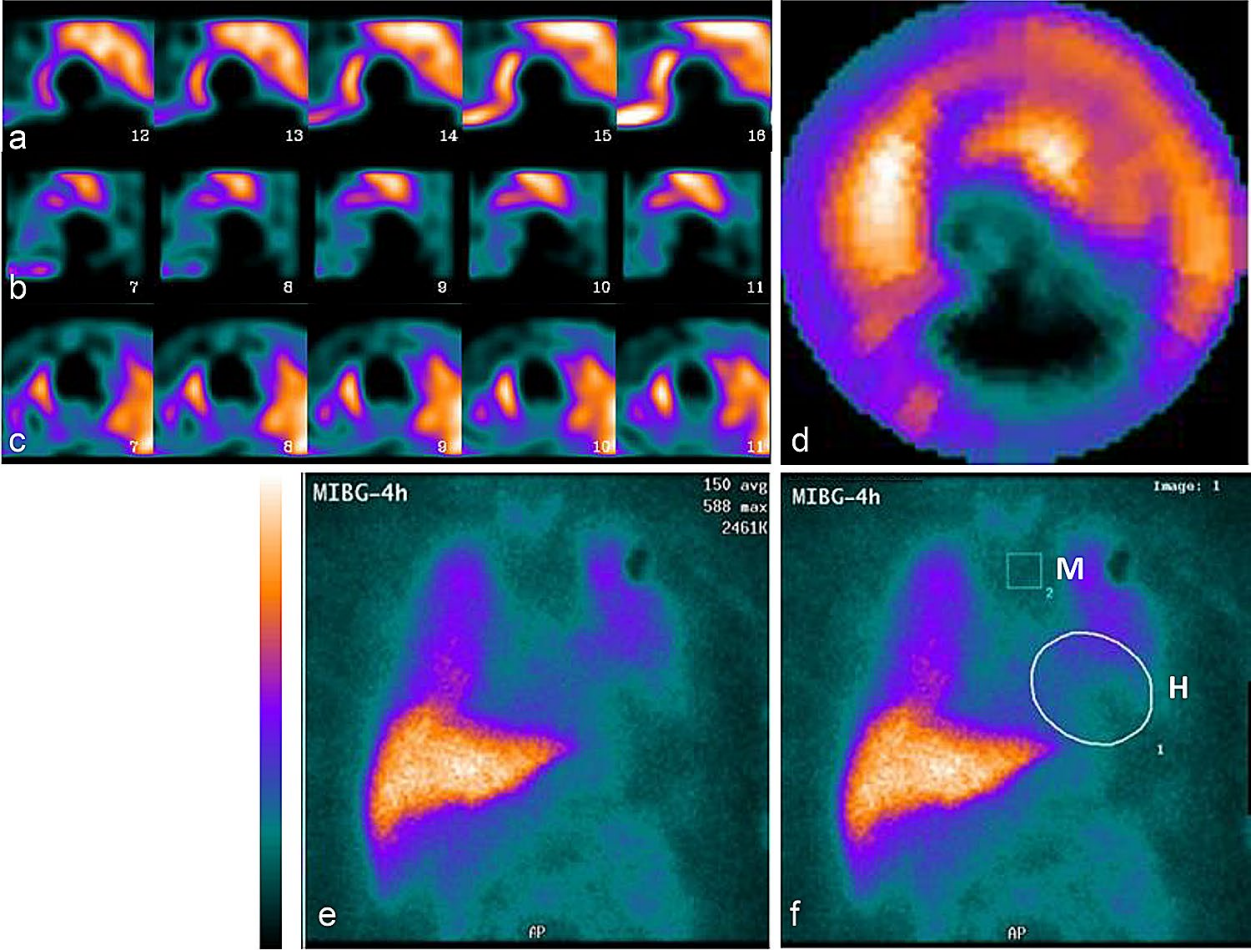
Example of late MIBG images in a nondiabetic patient with postinfarction heart failure. The quality of SPECT data was classified as “unacceptable” and the assessment of SPECT studies was precluded because of extremely high lung uptake non-separating from anterior, anterolateral and lateral myocardial walls (**a**—short axis, **b**—vertical long axis, **c**—horizontal long-axis slices, **d**—polar map). Plain anterior planar image of the thorax (**e**) and the image with ROI placed over the heart H and mediastinum M (**f**). Planar heart-to-mediastinum ratio HMR was calculated as 1.55 and was below average value of 1.58 in the studied group

In 13 of 50 patients (26%, CI_0.95_ < 16–40 >), with a mean HMR value of 1.48 ± 0.27 from planar studies, the quality of the SPECT images was “unacceptable” because of the very high extracardiac uptake of MIBG that was contiguous to the anterior, anterolateral or lateral LV walls (lung uptake in 7 patients), the inferior wall (2 patients) or the anterior and inferior walls (4 patients), so that the assessments of the SPECT studies were precluded (the group SPECTnonquant, Fig. 1). The categorization of the SPECT images as “unacceptable” in all the 13 patients was determined independently by each observer.

**Fig. 2:**
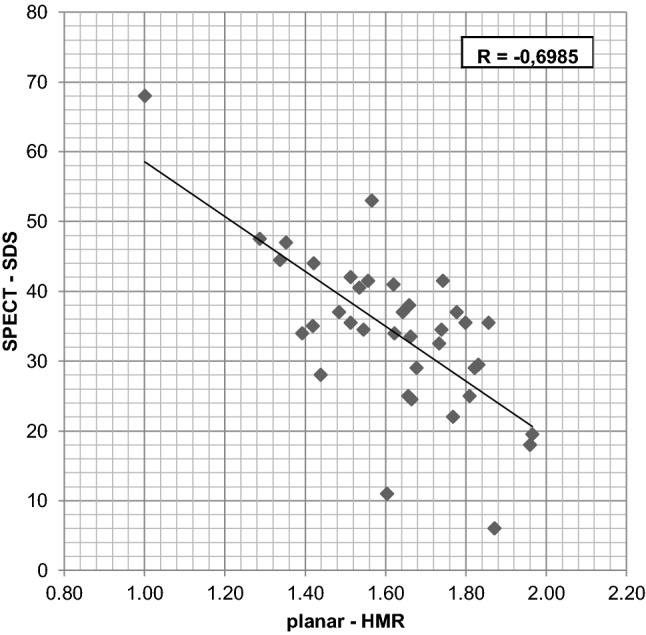
The relationship between heart-to-mediastinum ratio HMR from late planar image and the summed defect score SDS from late SPECT image

**Fig. 3:**
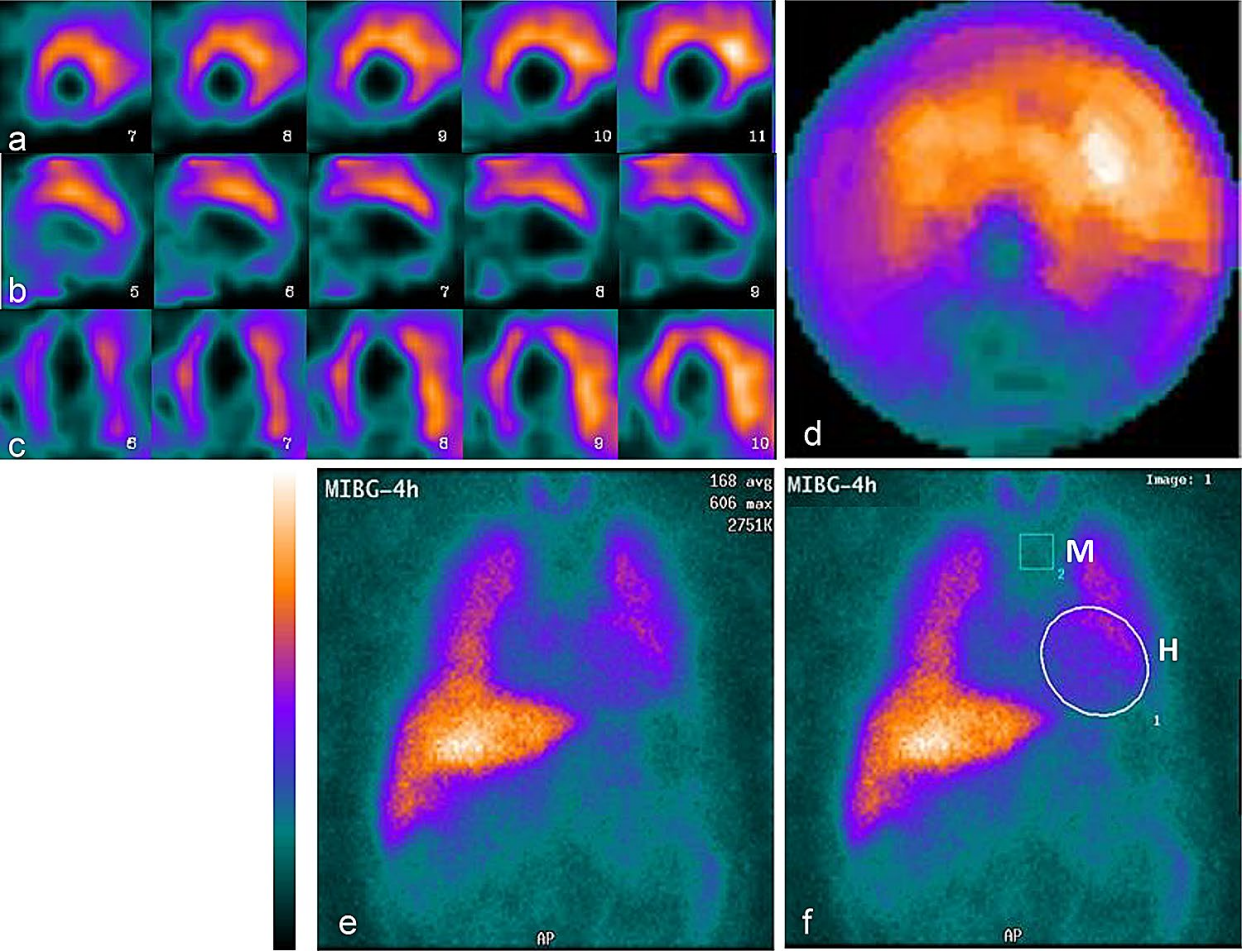
Example of late MIBG images in a nondiabetic patient with postinfarction heart failure. The quality of SPECT data was classified as “low”, presenting high extracardiac uptake interfering with uptake in anterior and anterolateral myocardial walls. Planar heart-to-mediastinum ratio HMR was calculated as 1.82 and was above average value of 1.58 in the studied group. Layout of images as in Fig. 1

**Fig. 4:**
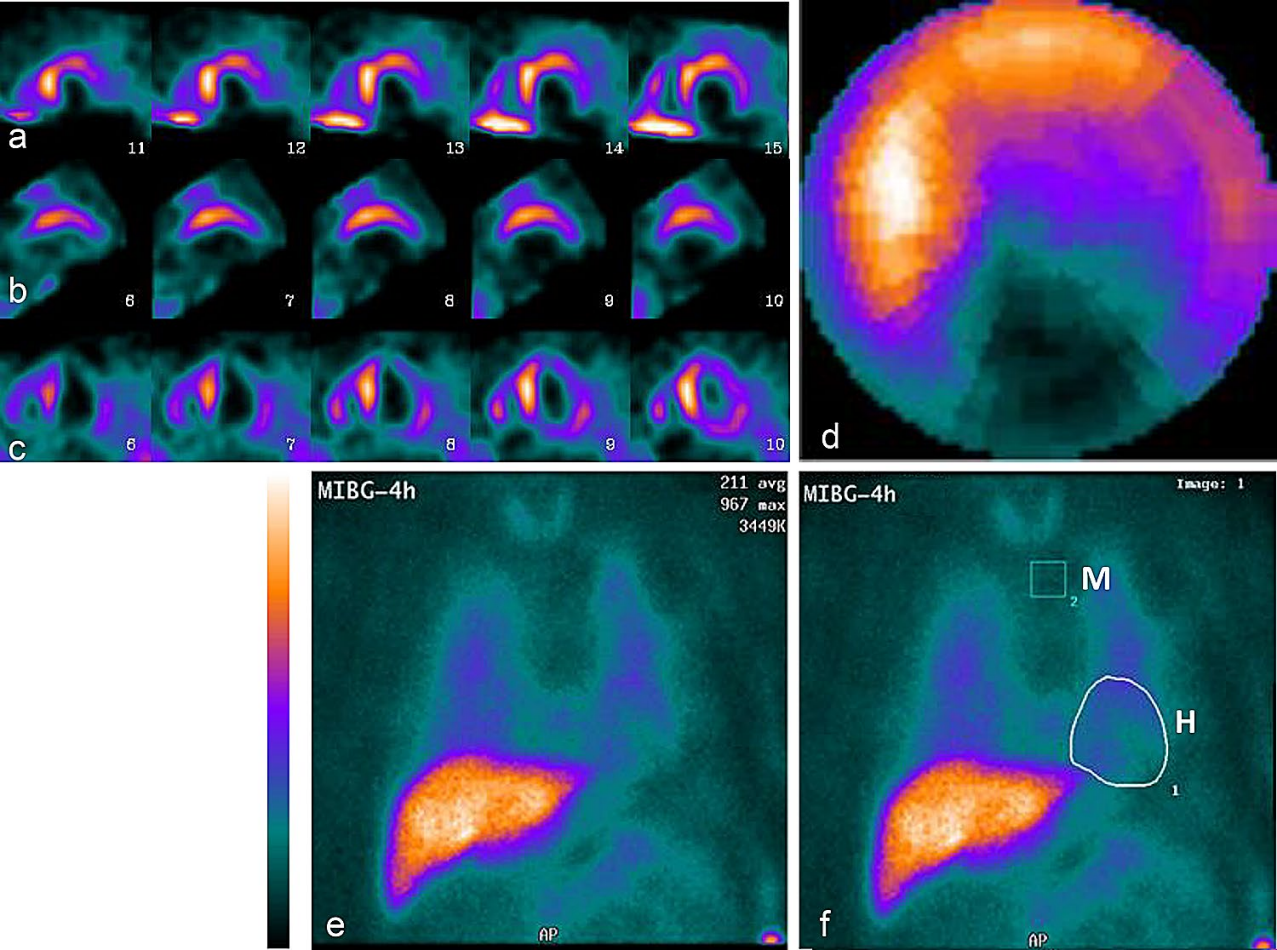
Example of late MIBG images in a nondiabetic patient with postinfarction heart failure. The quality of SPECT data was classified as “high”. Planar heart-to-mediastinum ratio HMR was calculated as 1.90 and was above average value of 1.58 in the studied group. Layout of images as in Fig. 1

In 37 patients submitted to the semiquantitative SPECT assessment (the group SPECTquant) with a mean HMR value of 1.62 ± 0.20 from planar studies, the SDS values ranged from 6 to 68 (mean of 34 ± 11, i.e., 50 ± 16% of the LV volume was denervated) (Table 2). The negative correlation between the SDS and HMR values was medium–strong (*R* = − 0.699, *p* < 0.0001) (Fig. 2). However, in 12 of those 37 patients, the quality of SPECT images was “low” and the assessments of the studies were equivocal in the opinion of at least one observer, because of the high extracardiac uptake that interfered with signals in the anterior, anterolateral or lateral LV walls (lung uptake in 5 patients), in the inferior or inferoseptal walls (4 patients), or in the anterior and inferior walls (3 patients). In addition, the assessment of SPECT image of one patient with no MIBG uptake in the heart was determined to be equivocal; in total, 13 of patients were qualified to the group SPECTlow (Fig. 3). The rest of the group SPECTquant consisted of 24 patients in which the quality of SPECT images was “high” in the opinion of both observers (the group SPECThigh, Fig. 4).

Altogether, in 26 of 50 patients (52%, CI_0.95_ < 38–65 >), the SPECT image quality was “unacceptable” (*n* = 13, SPECTnonquant) or “low” (*n* = 13, SPECTlow). A comparison of the extremes, the groups SPECTnonquant and SPECThigh, is presented in Table 3. The physical parameters that account for the quality of the SPECT images—weight and height of the patient and total dose—were similar. The average age of the patients in both groups was similar. The average values of the acknowledged markers of prognosis in HF patients (HMR and LVEF) were lower in the group SPECTnonquant relative to the group SPECThigh: HMR was borderline lower and LVEF was significantly lower. The number of patients with the device already implanted at the time of evaluation was 5 of 13 patients in the group SPECTnonquant (38%, CI_0.95_ < 17–65 >) and 16 of 24 in the group SPECThigh (67%, CI_0.95_ < 46–82 >), and the differences in their proportions were nonsignificant. These findings confirm the assumption that for research studies, MIBG imaging can be utilized shortly before, as well as shortly after implantation, as this intervention probably does not deteriorate the CANS.

**Table 3:** Characteristics of groups SPECTnonquant (*n* = 13, unacceptable quality of SPECT images) and SPECThigh (*n* = 24, high quality of SPECT images)

Characteristics	Group SPECTnonquant	Group SPECThigh	*p*
Body weight (kg)	75 ± 11	84 ± 15	NS
Height (cm)	171 ± 5	172 ± 8	NS
Total administered activity (MBq)	339 ± 30	333 ± 57	NS
Age (years)	68 ± 9	67 ± 8	NS
HMR	1.49 ± 0.27	1.63 ± 0.18	*p* = 0.051
LVEF (%)	25 ± 4	29 ± 6	*p* = 0.035

Values are means ± SD

*HMR* heart-to-mediastinum ratio, *LVEF* left ventricular ejection fraction, *SPECT* single-photon emission computed tomography

The planar images were interpretable in all the 50 patients. Reproducibility of H/M from planar images was high: intra-observer and inter-observer CV% values were 2.8% and 3.2%, respectively, with narrow 95% confidence intervals and with upper limits of the intervals below 5%. Reproducibility of SDS from SPECT images was lower: intra-observer and inter-observer CV% values were 9.4% and 10.5% (Table 4).

**Table 4:** Intra- and inter-observer reproducibility of count densities in manually drawn regions of interest (H, M), uptake ratio (H/M) and visual SPECT scoring (SDS)

	Intra-observer reproducibility^a^		Inter-observer reproducibility^b^		Number of patients
	CV (%)	95% confidence interval for CV (%)	CV (%)	95% confidence interval for CV (%)	
Planar (3.6 h imaging)					50
H	0.9	0.7–1.1	2.8	0–4.4	
M	3.2	2.4–3.8	2.9	0–4.4	
H/M	2.8	2.0–3.4	3.2	1.0–4.3	
SPECT (4 h imaging)					37
SDS	9.4	3.1–12.9	10.5	0–15.1	

*CV* coefficient of variation [26],* H* heart, *M* mediastinum, *SDS* summed defect score, *SPECT* single-photon emission computed tomography, *HMR* heart-to-mediastinum ratio, *MIBG* [^123^I]I-metaiodobenzylguanidine

^a^From 2 observations (observation 1 and observation 2) for Observer 1

^b^From observation 1 for Observer 1 and single observation for Observer 2

Approximately half (24/50, 48%) of the studied population presented good quality (group SPECThigh). The quality of SPECT images was connected with LVEF values: mean LVEF was significantly higher in SPECThigh (Table 3) and LVEF > 30 (and ≤ 35%) was observed in 7/24 (29%) cases of SPECThigh, in 8/37 (22%) cases of SPECTquant, and in none case of SPECTnonquant. The quality of SPECT images was also connected with HMR values from planar images: mean HMR was borderline lower in SPECTnonquant than in SPECThigh (Table 3), the lowest HMR values below 1.29 were observed only in cases of SPECTnonquant (3/13, 23%), and in SPECTquant group the medium–strong negative correlation between the SDS and HMR values was observed (Fig. 2).

In addition, consensus qualitative assessment of planar MIBG images corresponding to different groups of qualitative SPECT results showed liver uptake much higher than lung and heart uptake in all the 50 patients. In SPECTnonquant group, left lung uptake (Lung) in planar imaging was higher than heart uptake (Heart) in 10/13 (77%, CI_0.95_ < 49–92 >) and comparable to Heart in 3/13 (23%, CI_0.95_ < 8–51 >) patients. In SPECTlow, Lung was higher than Heart in 2/13 (15%, CI_0.95_ < 4–43 >), comparable to Heart in 9/13 (69%, CI_0.95_ < 42–88 >) and lower than Heart in 2/13 (15%, CI_0.95_ < 4–43 >) patients. In SPECThigh, Lung was higher than Heart in 5/24 (21%, CI_0.95_ < 9–41 >), comparable to Heart in 15/24 (63%, CI_0.95_ < 42–79 >) and lower than Heart in 4/24 (17%, CI_0.95_ < 7–36 >) patients. Altogether, in SPECTquant group, Lung was higher than Heart in 7/37 (19%, CI_0.95_ < 9–35 >) and comparable or lower in 30/37 (81%, CI_0.95_ < 65–91 >) patients; the proportions were significantly different than in SPECTnonquant group.

Early phase SPECT images had been obtained (33 ± 6 min post-MIBG injection) in 43 patients. The results of comparison of early and late SPECT assessment were as follows. (1) ‘Unacceptable’ image quality was observed in SPECT-late and SPECT-early in the same 12 of 43 (28%) patients; assessment was precluded in the anterior, anterolateral or lateral LV wall (6 patients), in the inferior wall (2 patients), or in the anterior and inferior walls (4 patients). (2) In SPECTquant studies, mean SDS-late (33 ± 9, range 12–48) was insignificantly higher than SDS-early (29 ± 9, range 11–44). (3) ‘Low’ image quality, because of high interfering extracardiac activities, was observed in SPECT-late and SPECT-early in the same 10 of 43 (23%) patients; assessment was equivocal in the anterior, anterolateral or lateral LV wall (3 patients), in the inferior or inferoseptal wall (4 patients), or in the anterior and inferior walls (3 patients). Three patients had exactly the same quality of early and late images (activity under inferior wall in 2 patients; activity under inferior wall and over anterior wall in 1 patient). In 2 patients, in late studies there was higher activity under inferior or inferoseptal wall. In 3 patients, in early studies, there was higher activity over anterior or anterolateral wall. In 2 patients, there was higher activity under inferior wall in late study and over anterolateral wall in early study.

## Discussion

In our study, it was determined that one-fourth of the MIBG SPECT images in the postinfarction HF patients could not be clinically assessed, and that another one-fourth was not of sufficient quality to provide an unequivocal conclusion. In patients that underwent a semiquantitative assessment, a significant, negative linear correlation between the HMR and SDS values was present (the lower global uptake in the planar study, the bigger defect score in SPECT). The linear correlation between HMR and SDS values shows that SDS values collected from SPECTquant cases allowing regional investigation (74% of our population), can be treated as an index of global cardiac adrenergic activity (or, more precisely, global denervation/dysinnervation). That approach was proposed by Boogers et al. in the SPECT study on predictive value of sympathetic nerve imaging on the occurrence of arrhythmias [8]. For obvious reason, that interpretation does not encompass patients with “unacceptable” SPECT quality (and unavailable SDS values). However, some of our results support the hypothesis about MIBG SPECT imaging as a technique for global cardiac adrenergic activity assessment also in this group: the mean values of HMR and LVEF were lower than in the group with a “high” image quality. These results confirm that the poor quality of the SPECT images is associated with a lower amount of global cardiac MIBG uptake (which reflects global impaired activity of the CANS), and with a lower global LV contractility. Thus, (1) the low cardiac MIBG uptake; (2) extracardiac activities that are equal to, or exceeding, the activity of the heart walls, as well as (3) known technical limitations (the limited spatial resolution of the SPECT technique and the construction of collimators not fully discriminating against detection of scattered photons from neighboring organs, especially LEHR collimators, which are accepted for ^123^I-imaging), result in the images where the layers of the heart are not distinguishable from the layers of contiguous structures in the patients with severe ischemic HF. The “overlapping” effect is caused predominantly by the lung uptake of MIBG (because of immediate vicinity of lungs and heart in every patient) and to a lesser extent by the uptake in the organs of gastrointestinal tract (which separate from the heart in part of the patients), although physiological heart-to-liver ratio is lower than heart-to-lung ratio [1]. The problem may be even more pronounced in diabetic patients, as the lung uptake of MIBG is increased in DM patients with sympathetic nervous dysfunction [27]. On the other hand, the problem with the activities from GI tract will be probably further reduced in diabetic patients, as HF patients with DM present lower liver uptake than patients without DM [28].

As the patients with “unacceptable” quality SPECT images were not older than the patients that underwent a semiquantitative assessment, the results of the study cannot be attributed to a lower MIBG uptake reported in the elderly [29].

MIBG SPECT imaging has been proposed for: (1) improved ability to determine the global state of cardiac innervation, because it is able to overcome the superposition of non-cardiac structures that are present in the planar technique [5, 6] and (2) its ability to provide regional information that is not available from planar images [30]. In some of the review papers, it has been mentioned that SPECT images of patients with advanced HF can be difficult to interpret [30, 31]. However, in the original papers, hardly sometimes the proportion of uninterpretable or equivocal SPECT images was presented, and to our knowledge, there is no paper that assesses that proportion in a population of patients with postinfarction HF and no DM.

In two important original papers, it can be found the information on the high number of unacceptable or low quality of MIBG SPECT images. In the work of Bax et al., concerning 50 patients with LV dysfunction and previous myocardial infarction, three observers considered all of the MIBG SPECT images as interpretable, but only 38–94% were determined to be of optimal quality [7]. Similar to our study, LEHR collimators and FBP reconstruction were utilized. If our criteria for quality were applied, one of the experienced observers would have judged that in 16/50 of patients, the quality of the SPECT data was “unacceptable”. His results would be even worse to ours (13/50 of patients), possibly on account of 32% of DM patients included. The recent large study (ADMIRE-HF Study) reported on the potential utility of MIBG SPECT images that were evaluated in 621 ischemic HF patients [32]. For data acquisition, LEHR collimators were utilized, and for image reconstruction, the optimized iterative method was applied. If our criteria for assessment were applied, similar to our results the “unacceptable” quality label would be assigned to 150 studies (24%) and “low” quality label—to another 156 studies (25%). Although the study included patients with DM, the proportion of SPECT images of “unacceptable” and “low” quality would not be higher than in our population—possibly because of the inclusion of some patients that did not have a myocardial infarction and/or because of the optimized iterative reconstruction.

Yamamoto et al. reported on 7 of 80 HF patients (9%) excluded from the analysis of regional washout rate in SPECT (LEHR collimators and FBP reconstruction) because of too low MIBG uptake in 2 patients and overlying extracardiac (lung and liver) accumulation interfering the cardiac visibility in 5 patients [33].

In the other papers, the issue of low quality of MIBG SPECT studies performed with LEHR collimators and FBP reconstruction is not raised in HF patients. The study by Boogers et al. included 116 patients enrolled prior to ICD implantation and only three MIBG SPECT images were noninterpretable [8]. The overall group was heterogeneous—with 14% of patients that had DM, 11% with an implanted ICD for secondary prevention (in whom greater deterioration of the CANS could be expected) and 26% with non-ischemic cardiomyopathy (in whom significantly smaller MIBG SPECT abnormalities, compared with postinfarction HF subjects were proved [34]). Pellegrino et al. demonstrated a high intra- and inter-observer reproducibility of SDS in late SPECT studies even at the lowest recommended dose (111 MBq) in 74 patients with HF and LV systolic dysfunction, with no recorded problems in interpreting the images [35]; 51% of patients had DM and 66% had ischemic HF. In the recent prospective single-center study, De Vincentis et al. addressed the usefulness of cardiac MIBG SPECT imaging in 170 HF patients referred for ICD implantation for primary (92%) or secondary prevention (59% of the patients had ischemic HF and 36% had DM) [36]. The authors do not mention the SPECT studies that were considered of suboptimal quality and showed an excellent inter-observer agreement for both early and late SDS values. In our study, the intra- and inter-observer reproducibility of SDS assessment in ischemic, postinfarction HF patients, without DM, qualified for ICD in primary prevention of SCD was approximately three times lower than reproducibility of H/M assessment from planar images. This was mainly a consequence of uncertainty in grading of MIBG uptake in the patients with “low” quality of SPECT images.

An improved method for assessment of low-count MIBG SPECT images has been proposed, by utilizing the SPECT/CT technique [34, 37]. Notwithstanding, the suggested circumferential approaches do not have the ability to correct interfering extracardiac MIBG uptake in the resulting polar maps, or to improve the regional visual assessment of the true distribution of MIBG in SPECT image slices.

As the specific changes of MIBG uptake and washout from liver and lungs were observed in HF patients [28, 38], early-phase imaging might provide better visual evaluation SPECT images graded as ‘unacceptable’ and/or ‘low’ quality in late phase imaging. Liver washout was calculated as much slower than lung washout and than myocardial washout in chronic HF [38]. Study of decay-corrected liver activity in patients with HF and without DM in the ADMIRE-HF population showed that late liver activity significantly increases [28]. Our studies do not prove the superiority of early SPECT imaging in nondiabetic postinfarction HF patients. ‘Unacceptable’ SPECT-late images always corresponded to ‘unacceptable’ SPECT-early images. The ‘low’ quality of SPECT-late images always concerned also SPECT-early images, most often with higher activity from lungs in early imaging and often with higher activity from liver in late imaging.

The very recent multicenter test–retest study showed that HMR derived from a planar acquisition, which is a strong predictor of outcomes in patients with stable class II–III HF and LVEF ≤ 35% [22], is a consistent and highly reproducible measurement (in a population including 55% of patients with ischemic HF etiology and 33% of patients with diabetes) [39]. That study, in parallel with the earlier studies (in populations including 63–66% of patients with ischemic HF and 13–51% of patients with DM) [35, 40], showed very low inter- and intra-observer variability of planar HMR assessment in HF patients. In our study, we confirmed high reproducibility of planar HMR late assessment in ischemic, postinfarction HF patients, without DM, qualified for ICD in primary prevention of SCD.

Assessing regional uptake of MIBG on tomographic images has been proposed basing on the idea that local abnormalities of sympathetic innervation create electrical instability predisposing to life-threatening arrhythmias, especially if such regions are perfused and viable [41]. Arising therefrom studies have been aimed to study a neuronal/perfusion mismatch responsible for denervation supersensitivity [7, 9, 31, 32]. Zhou et al. developed a method to measure scar and border zone and showed that MIBI uptake in the border zone predicted ventricular arrhythmias with a promising accuracy [9]. However, in the study by Bax et al., innervation–perfusion mismatch score was not predictive of inducible ventricular tachyarrhythmias [7]. Travin et al. also showed that the summed innervations/perfusion mismatch was not significant for predicting of arrhythmic events and concluded, that the presumption of a monotonic increase in arrhythmia risk with increasing SDS MIBG SPECT score may not be correct as ischemic HF patients with abnormalities of intermediate extent appear at highest risk [32]. All the cited results point out, that the clinical usefulness demands high-quality MIBG tomograms allowing unequivocal regional interpretation, possibly quantitative, like in protocols established for perfusion studies.In our study, although approximately half of the nondiabetic postinfarction HF patients showed unacceptable or poor SPECT quality (groups SPECTnonquant and SPECTlow), the other half (48%) presented good quality (group SPECThigh) and unequivocally visualized regional information, which is of prognostic importance, as both global and regional sympathetic denervation or dysinnervation predispose patients after myocardial infarction to ventricular arrhythmias [42].

Connecting the data available from our material before proceeding to SPECT studies, we received parameters of importance:

- HMR < 1.29 warranted SPECTnonquant
- LVEF > 30% warranted SPECTquant (although not necessary SPECThigh)
- left lung uptake intensity in planar images was higher than in heart in most patients with SPECTnonquant (77%, CI_0.95_ < 49–92 >) and nearly 4 times less frequent in patients SPECTquant (19%, CI_0.95_ < 9–35 >)
- liver uptake intensity in planar studies was always much higher than left lung and heart uptake.

Having the knowledge about the limited reproducibility of LVEF and HMR, it seems more appropriate to change the cutoff values into the criteria predisposing the patient to SPECTnonquant, which were:

- close to one value of HMR in planar imaging (weak uptake in a heart)
- left lung uptake intensity in planar images higher than heart uptake.

On the other hand, the criteria predisposing the patient to SPECTquant were:

- high values of LVEF from the range characterizing the patients qualified to ICD (i.e., close to 35%)
- left lung uptake intensity in planar images comparable to or lower than heart uptake.

### Study limitations

The study has some limitations. First, a relatively small number of patients is included. However, as a result of utilizing rigorous inclusion/exclusion criteria, the findings concern a uniform and unique group of 50 nondiabetic subjects with postinfarction HF, who are considered for device implantation for primary prevention of SCD. Second, LEHR collimators were utilized, which are optimized for the ^99m^Tc and not for the ^123^I photopeak energy. However, LEHR collimators are accepted and most often utilized for [^123^I]I-metaiodobenzylguanidine imaging [7, 8, 22, 32–36, 39]. Third, the SPECT data were reconstructed through FBP, which does not offer efficient compensation of attenuation and collimator response. Although FBP algorithm has been utilized in important studies [7, 8, 22, 33–36], iterative methods of reconstruction provide gains in both signal-to-noise ratio and reduction of artifacts [43]. However, despite state-of-art iterative reconstruction, the quality of the SPECT imaging in the ADMIRE-HF Study could be summarized similarly to our study [32].

### Conclusions

The utility of MIBG SPECT, at least with conventional imaging protocol, in the qualification of HF patients for an ICD is limited. In approximately half of the postinfarction HF patients, SPECT assessment of cardiac innervation can be impossible or equivocal, even without additional damage from diabetic cardiac neuropathy. The value of SPECT imaging is limited by low MIBG cardiac uptake and by comparatively high, interfering MIBG uptake in the neighboring structures (primarily, in the lungs). The criteria predisposing the patient to good-quality MIBG SPECT are: high values of LVEF from the range characterizing the patients qualified to ICD (i.e., close to 35%) and left lung uptake intensity in planar images comparable to or lower than heart uptake.
